# Supplementary material for: Stacking Model‐Based Classifiers for Dealing With Multiple Sets of Noisy Labels
Source: Biom J. 2025 Mar 12;67(2):e70042. doi: 10.1002/bimj.70042 (PMC11898607; doi:10.1002/bimj.70042)
Supplement: Supplementary file 1 — Supporting Information [file BIMJ-67-e70042-s001.zip › density-based-ensemble-model-main/competitors_real_data/netlab-master/netlab-master/help/gmmunpak.htm]

Netlab Reference Manual gmmunpak


# gmmunpak

## Purpose

Separates a vector of Gaussian mixture model parameters into its components.

## Synopsis

```
mix = gmmunpak(mix, p)
```

## Description

`mix = gmmunpak(mix, p)`
takes a GMM data structure `mix` and
a single row vector of parameters `p` and returns a mixture data structure
identical to the input `mix`, except that the mixing coefficients
`priors`, centres `centres` and covariances `covars`
(and, for PPCA, the lambdas and U (PCA sub-spaces)) are all set
to the corresponding elements of `p`.

## See Also

`gmm`, `gmmpak`

---

**Pages:**
Index


---

Copyright (c) Ian T Nabney (1996-9)
